# Genome-proteome system for prostate cancer

**DOI:** 10.1186/1878-5085-5-S1-A28

**Published:** 2014-02-11

**Authors:** Maxim N Peshkov

**Affiliations:** 1 Scientific Research Institute of Physical-Chemical medicine, Moscow, Russia

## 

Cancer of prostate is challenging medical issue which prevalence ranking in men among all oncologic diseases corresponds to the 4th most prevalent in Russia, 2^nd^-3^rd^ in West Europe and 1^st^ in USA since year 2007. The most promising approach follows the strategy of early detection of tumor progress prior to clinical manifestation in prostate gland. The PSA (prostate specific antigen) is used as the universal marker in clinical practice since 1979 year, however, with modest success. Hence, 40% of prostate cancer patients demonstrate normal levels of PSA (n<2,5ng/ml), and only 60% of the patients have higher levels of PSA (n>4,0 ng/ml). Consequently, the world research community further efforts to develop more specific and sensitive markers for early detection of pre-cancerous lesions in prostate gland. A promising tool might be “omics” and in particular “proteomics” technologies. Predictive, Preventive and Personalized medicine is considered as the medicine of XXI century with following requirements: risk assessment, disease specific profiles, early/predictive diagnostics, disease/treatment monitoring, reliable prognosis, and treatments tailored to the person.

## Scientific objectives

development of a genomic-proteomic system for early diagnosis and clinical monitoring of patients with prostate cancer as follows:

• To determine a genome-wide significant association between PSA level and single-nucleotide polymorphisms (SNPs) at loci;

• To define potential candidates for blood peptide's biomarker expression database, as the platform for the identification, quantification and validation of protein biomarker(s) during prostate cancer progression;

• To create a centralized prostate cancer urine peptide's biomarker expression database with add value for the blood biomarkers database;

• To analyze diagnostic accuracy of the prostate cancer biomarker candidates;

• To create innovative approaches for risk assessment, therapy monitoring and treatment management for this patient cohort.

**Table 1:** Types of prostate cancer specific alterations that can be potentially considered as biomarker candidates

Level of research	Research unit	Biomaterial	Detection technology
**Genome**	Polymorphism (`s)	Blood	PCR, Restriction analysis ets.

	Methylation status of GpC island	Blood	Methylation-specific PCR

**Transcriptome**	MicroRNAs (miRNAs)	Blood	MALDI-TOF based mini-sequencing

	Reduced mRNA editing	Blood	Reverse-Transcriptase-PCR

**Proteome**	Protein of plasma	Blood plasma	Multiple Reaction Monitoring, (MRM)

	Protein of urine	Urine	Multiple Reaction Monitoring, (MRM)

	Protein, peptide of tissue	Paraffin block (tissue)	Multiple Reaction Monitoring, (MRM) and Information Dependent Acquisition (IDA)

**Metabilites**	Protein of plasma	Blood plasma	Multiple Reaction Monitoring, (MRM)

	Protein of urine	Urine	Multiple Reaction Monitoring, (MRM)

**Macro-micro tissue analysis (morphological diagnosis)**	Tissue	Biopsy material	Microscopy, immunohistochemistry

## Methodology

With altogether over 1000 prostate cancer patients a robust database has been created in the study for follow-up molecular biological analysis. To estimate a genome-wide significant association between PSA level and single-nucleotide polymorphisms (SNPs) at loci, MALDI-TOF based mini-sequencing of blood samples is utilized.

## Outlook

The sequencing data will be correlated with other parameters such as individual PSA levels, cancer stage and tumor aggressiveness. In the next steps, blood and urine profiles will be analyzed at the level of proteins. Multi-factorial analyses are expected to reveal correlated between individual parameters. Creation of the genome-transcriptome-proteome patterns is the promising approach to establish early detection, predictive diagnostics, reliable prognostics and improved management of prostate cancer.

